# Effects of exogenous enzymes and application method on nutrient intake, digestibility and growth performance of Pelibuey lambs

**DOI:** 10.1186/s40064-016-3075-7

**Published:** 2016-08-23

**Authors:** Daniel López-Aguirre, Javier Hernández-Meléndez, Rolando Rojo, Fernando Sánchez-Dávila, Nicolás López-Villalobos, Abdel-Fattah Z. M. Salem, Juan Carlos Martínez-González, José Fernando Vázquez-Armijo, Salomón Ruíz

**Affiliations:** 1 Centro Universitario UAEM Temascaltepec, Universidad Autónoma del Estado de México, 51300 Temascaltepec, México Mexico; 2 Facultad de Ingeniería y Ciencias, Universidad Autónoma de Tamaulipas, 87000 Ciudad Victoria, Tamaulipas Mexico; 3 Facultad de Agronomía, Universidad Autónoma de Nuevo León, 65500 Escobedo, Nuevo León Mexico; 4 Institute of Veterinary, Animal and Biomedical Sciences, Massey University, Palmerston North, 4442 New Zealand; 5 Facultad de Medicina Veterinaria y Zootecnia, Universidad Autónoma del Estado de México, 50200 El Cerrillo Piedras Blancas, México Mexico

**Keywords:** Application method, Digestibility, Exogenous enzymes, Lambs, Performance

## Abstract

Pelibuey sheep is the main breed in the tropical and subtropical regions of Mexico, and high demand of sheep meat has favored the finishing of lambs in feedlots with diets containing high levels of grains. The objective of this study was to evaluate the effects of exogenous enzymes (EE) and application method on nutrient intake and digestibility and performance of growing Pelibuey lambs. Treatments were based on comparison of two different methods of adding an enzyme product (sprayed on the total mixed ration or applied orally to the lambs) versus control treatment (no added enzyme). Twenty-one Pelibuey lambs, weighing 15.7 kg (SD = 1.8 kg) initial body weight, were individually housed in shaded pens and assigned randomly to one of the three enzyme treatments. At the end of study (lasting for 45 days), three lambs from each treatment were randomly selected and adapted to a pants and harness designed for fecal collection to measure nutrient digestibilities. Total body gain and average daily gain were affected (*P* < 0.05) by supplemental EE. The application method of EE had significant (*P* < 0.05) effect on FCE and FCR, but no effects were observed on nutrient intake. Supplemental EE did improve (*P* < 0.05) the digestibilities of dry matter, organic matter, neutral and acid detergent fiber, but no differences were observed in crude protein digestibility. The application method of EE had significant (*P* < 0.05) effect on the digestibility of acid detergent fiber. Supplemental EE can improve body weight gain and nutrient digestibilities without affecting nutrient intake in Pelibuey lambs, but the results of feed conversion efficiency and acid detergent fiber digestibility depend on the application method used of the EE.

## Background

Pelibuey sheep is the main breed in the tropical and subtropical regions of Mexico, probably due to exceptional fecundity and adaptability to heat, humidity, parasites, scarcity of feed and other harsh environment conditions. High demand of sheep meat has favored the exploitation of Pelibuey sheep in the northeast dry tropic of México with breeding stock under grazing conditions and growing lambs finished in feedlots with diets containing high levels of grains. Inclusion of high levels of grain in the diet for ruminants has been associated with declining rates of fiber digestion due to lower fibrolytic activity in the rumen (Martin and Michalet-Doreau 1995; Noziere et al. 1996) and an apparent microbial preference for non-structural carbohydrates (Mould and Ørskov 1983). This may create conditions in which supplementary exogenous enzymes (EE) can have beneficial effects.

The beneficial impact of addition of EE depends on several factors such as diet composition, type of enzyme preparation, enzyme stability, specific enzyme activities, amount of enzyme added and application method (Yang et al. 2000; Morgavi et al. 2001; Wallace et al. 2001). Applying fibrolytic exogenous enzymes in a liquid form to feeds prior to consumption can have a positive effect on animal performance (Rode et al. 1999; Yang et al. 2000). The close association of enzymes with feed may enable some form of pre-ingestive attack of the enzymes upon the plant fiber and enhance binding of the enzymes to the feed. Kung et al. (2000) emphasized the importance of interaction time, since enzyme addition to feeds may create a stable enzyme-feed complex that protects free enzymes from proteolysis in the rumen, but other authors have indicated an alteration in fiber structure, which would stimulate microbial colonization (Nsereko et al. 2000). In contrast, direct infusion of enzyme into the rumen is far less effective or can even reduce digestibility of forages compared with application of liquid enzyme to substrate (Lewis et al. 1996). Yang et al. (2000) reported increased milk production and digestibility of the diet when enzymes were added to the concentrate portion of a dairy cow diet, but not when they were added directly to total mixed ration. The objective of this study was to evaluate the effect of cellulases exogenous enzymes and application method on nutrient digestibilities and performance of growing Pelibuey lambs.

## Methods

### Animals and diets

The study was conducted at an experimental farm of Facultad de Ingeniería y Ciencias-Universidad Autónoma de Tamaulipas, located in northeast Mexico (23°56′N, 99°06′W), at 190 m above sea level. The climate is considered dry tropic, which is semi-arid and sub-humid, with summer rains and sporadic winter rains. The average annual temperature is 23 °C with a total annual rainfall of 800 mm. All procedures involving the animals followed the international guiding principles.

Twenty-one Pelibuey lambs were selected two weeks after weaning (at about 60 days of age). Lambs (average initial body weight, BW ± SD, 15.7 ± 1.8 kg) were individually housed in shaded pens (1.5 × 0.75 m) and assigned randomly to one of three enzyme treatments in a completely randomized experimental design. One week before the starting of the study, animals were treated for internal and external parasites, gradually introduced to the experimental diet and adapted to the individual pens. A total mixed ration (TMR, 130 g CP/kg DM) was formulated to meet the nutritional requirements for growing lambs (NRC 2007) (Table 1). TMR diet was offered once a day at 08:00 h and all animals had free access to clean water throughout the experiment.

**Table 1:** Ingredients and chemical composition (g/kg DM) of basal diet of buffel grass fed to growing male Pelibuey lambs

Ingredient	Basal diet	Buffel grass
Sorghum grain	408	
Soybean meal	187	
Buffel grass hay	300	
Molasses cane	80	
Mineral premix^a^	25	
Chemical composition		
Crude protein	12.62	3.31
Metabolizable energy (Mcal/kg)	2.92	1.10
Neutral detergent fiber	434	805
Acid detergent fiber	179	511
Hemicellulose	255	294
Organic matter	901	875

^a^Composition per 1 kg contained (vitamin A 2,000,000 IU, vitamin D3 40,000 IU, vitamin E 400 mg, Mn 12.80 g, Zn 9.00 g, I 1.56 g, Fe 6.42 g, Cu1.60 g, Co 50 mg, Se 32 mg plus antioxidant)

### Enzyme product and application methods

Treatments were based on two different methods of adding an enzyme product to the diet, both provided at the same daily dose rate. The exogenous enzyme product used was Dyadic^®^ Cellulase PLUS, which is a concentrated liquid acid cellulase (E.C. 3.2.1.4) enzyme produced from *Trichoderma longibrachiatum* (formerly *Trichoderma reesei*) containing 30,000–36,000 U/g of Cellulase and 7500 to 10,000 U/g of beta-glucanase activity with additional side activities: xylanase, pectinase, mannanase, xyloglucanase, laminarase, β-glucosidase, β-xylosidase, α-L-arabinofuranosidase, amylase, protease. The daily dose of the enzyme product was measured per kilogram of DM intake. The enzyme treatments were:

1. TMR fed *ad libitum* with no added enzyme (Control).
2. Control TMR diet plus enzyme product (TMR-E). 1 ml of enzyme product was diluted in 10 ml of distilled water, and 10 ml of diluted enzyme were added per kg of TMR DM. This was equivalent to 1 ml of enzyme product per kg of TMR DM. Diluted enzyme solution was sprayed on the TMR and mixed daily 24 h before morning feeding.
3. Control TMR plus enzyme product applied orally (Oral-E). The enzyme product was diluted as explained in the TMR-E treatment but the solution was given orally to each lamb using a syringe 1 h before the morning feeding. The daily dose of the diluted enzyme product was applied at 10 ml per kg of TMR DM. The dosage was calculated according to the dry matter intake recorded the previous day.

### Animal measurements

The amounts of TMR offered and refused were recorded daily for each lamb and were adjusted to ensure *ad libitum* consumption (refusal of about 10 % of DM intake). For each lamb, individual refusals of feed were weighed daily during the experimental period and stored at −20 °C until further analysis. Offered and refusal feed samples were analyzed for DM and other nutrients in order to evaluate daily nutrient intake. Lambs were weighed at the beginning of the experiment and subsequent weights were measured every 15 days before the morning feeding. Then, total gain and average daily gain (ADG) for lambs were calculated by subtraction of the initial from final BW and then dividing by duration of the study (51 days). Feed conversion ratio (FCR = DM intake/ADG) and feed conversion efficiency (FCE = ADG/DM intake) were also calculated.

### Nutrient digestibility

At the end of the study, three lambs of similar BW and DM intake from each treatment were randomly selected and adapted to a pants and harness designed for total fecal collection to measure nutrient digestibilities. Lambs had 2 days of an adaptation period to pants and harness followed by 5 days of sample collection. Feed intake and refusals were recorded and sampled for further analysis. Daily fecal output was collected, weighed, and recorded. A sub-sample (10 % of total feces) was stored (−18 °C) for latter analysis.

### Analytical methods

TMR diet and refusal samples were dried at 55 °C in a forced-air oven to reach a constant weight, air equilibrated and ground to pass through 1 mm sieve. Samples of TMR diet offered and feed refusals were analyzed for dry matter (DM; 105 °C in forced-air oven for 24 h), organic matter (OM; weight loss upon ashing at 550 °C for 6 h) and nitrogen (N; Kjeldahl procedure) according to AOAC (1990). The crude protein (CP) content was calculated by multiplying N by 6.25. Neutral detergent fiber (NDF; with heat stable α-amylase and Na sulfite) and acid detergent fiber (ADF) content (Van Soest et al. 1991) were analyzed using the ANKOM F-57 filter bags in an ANKOM200 fiber analyzer (ANKOM Technolgy, Macedon, NY USA). Values for NDF and ADF were not corrected for ash contents. Hemicellulose content was calculated from the difference between NDF and ADF. Samples of the dried feces, offered and refused feed obtained from the digestibility study were analyzed for DM, OM, CP, NDF and ADF to calculate nutrient digestibilities.

### Statistical analysis

Data were analyzed as a randomized complete design with three treatments (Control, TMR-E and Oral-E) with seven repetitions per treatment (three in case of digestibility traits) using the GLM procedure of the statistical package SAS version 9.2 (SAS Institute, Cary, NC, USA). The linear model included the fixed effect of treatment. Initial BW was included as a covariate for the BW data. Orthogonal contrasts were used to determine effect of enzyme addition (contrast 1, Control vs TMR-E and Oral-E) and application method (contrast 2, Oral-E vs TMR-E) on all dependent variables. Least squares means and standard errors for each treatment were obtained and used for multiple mean comparisons using the LSD test as implemented in the GLM procedure.

## Results

Effects of supplemental EE on lamb performance and nutrient intakes of growing Pelibuey male lambs are presented in Table 2. As intended by design, initial BW was similar for both treatment groups. Addition of EE had no effect on lamb’s intake of the basal diet in terms of DM, OM, CP, NDF and ADF. Total gain and ADG were affected (*P* < 0.05) by supplemental EE, but no effects were observed on FCE and FCR. The application method of EE (Oral-E vs TMR-E) had significant (*P* < 0.05) effect on FCE and FCR, but no effects were observed on nutrient intake.

**Table 2:** Effect of supplemental exogenous enzymes and application method on the productive performance and nutrient intake of growing Pelibuey male lambs fed a diet containing buffel grass

	Control	Application method^a^		SEM	Contrast^b^ (P values):	
		Oral-E	TMR-E		C1	C2
N	7	7	7			
Initial BW (kg)	15.24	15.10	16.71	0.66	0.4264	0.1039
Final BW (kg)	23.62	24.32	24.62	0.26	0.0144	0.4415
Total gain (kg)	7.93	8.63	8.93	0.26	0.0144	0.4415
ADG^c^ (g/d)	193	210	218	6.3	0.0144	0.4415
Nutrient intake, g/d						
DM	1029	1120	1084	44.4	0.1041	0.4770
OM	928	963	976	22.4	0.3999	0.8169
CP	130	135	137	3.13	0.3989	0.8173
NDF	447	464	470	10.79	0.4033	0.8163
ADF	184	191	194	4.45	0.4005	0.8226
Feed conversion efficiency^d^	184.8^x^	182.8^x^	208.0^y^	6.5	0.1969	0.0166
Feed conversion ratio^d^	5.46^x^	5.51^x^	4.86^y^	0.18	0.2398	0.0283

^a^Basal diet supplemented with 0 (Control, no enzyme) or with 1 ml of supplemental exogenous enzyme per kg DM of diet applied orally to each lamb 1 h before morning feeding (Oral-E) or sprayed on the diet (TMR-E) 24 h before morning feeding

^b^C1, Control versus Oral-E and TMR-E; C2, Oral-E versus TMR-E

^c^Average daily gain calculated as (final BW–initial BW)/duration of study

^d^Feed conversation efficiency = ADG/DM intake (g body weight gain/kg DM); Feed conversion ratio = DM intake/ADG (kg DM/kg body weight gain)

^x,y^Means within the same row with different superscript are significantly different (*P* < 0.05)

Addition of EE did improve (*P* < 0.05) the digestibilities of DM, OM, NDF and ADF, but did not improve the CP digestibility of the diet used for the lambs in this study (Table 3). Additionally, the application method of EE had significant (*P* < 0.05) effect on ADF digestibility; the enzyme applied directly on the diet resulted in the highest ADF digestibility (Table 3).

**Table 3:** Effect of supplemental exogenous enzymes and application method on nutrient digestibility of growing Pelibuey male lambs fed a diet containing buffel grass

Item	Control	Application method^a^		SEM	Contrast^b^ (P values):	
		Oral-E	TMR-E		C1	C2
n	3	3	3			
Digestibility (g/kg)						
DM	742	796	793	9.52	0.0017	0.7968
OM	768	818	817	9.18	0.0020	0.9537
CP	754	775	780	6.56	0.1223	0.7483
NDF	667	715	724	10.78	0.0122	0.6063
ADF	349	365	440	15.78	0.0254	0.0116

^a^Basal diet supplemented with 0 (Control, no enzyme) or with 1 ml of supplemental exogenous enzyme per kg DM of diet applied orally to each lamb 1 h before morning feeding (Oral-E) or sprayed on the diet (TMR-E) 24 h before morning feeding

^b^C1, control versus Oral-E and TMR-E; C2, Oral-E versus TMR-E

## Discussion

Research on effects of adding EE on nutritive value of the diet and animal performance are not consistent. In this study, the addition of the EE product did not improve intake of nutrients, but ADG of the lambs was improved possibly due to the improvement of nutrient digestibility. Previous research reported that application of EE preparations of different compositions improved DM intake and growth performance of lambs (Cruywagen and van Zyl 2008), goat kids (Titi 2003) and beef steers (Beauchemin et al. 1999; McAllister et al. 1999) and nutrient digestibility and milk production in dairy cattle (Yang et al. 1999; Gado et al. 2009). In contrast, other studies have reported that EE supplementation failed to improve DM intake and growth performance of lambs (McAllister et al. 2000; Mora-Jaimes et al. 2002; Rojo et al. 2005; Miller et al. 2008a; Awawdeh and Obeidat 2011) and beef steers (Krueger et al. 2008; Miller et al. 2008b).

This study found that the EE product improved the digestibility of DM, OM, NDF and ADF, but not the digestibility of CP of this diet for Pelibuey lambs. These results agree with most the published literature reporting increased total tract digestibilities of DM, OM or both, following treatment with a fibrolytic enzyme mixture (Rode et al. 1999; Yang et al. 1999; Beauchemin et al. 2000). Specifically, in a diet for lambs, Titi and Tabbaa (2004) reported that digestibility of nutrients was improved with the addition of cellulase enzyme, but the digestibility of CP was not affected.

It has been suggested that improved digestibility caused by EE treatment of feed might be related to improved microbial colonization (Yang et al. 1999; Nsereko et al. 2002) or both improved colonization and increased activity of the EE within the rumen (Beauchemin et al. 2000). This view is similar to previous hypotheses that exogenous enzymes increased fibrolytic activity due to increased numbers of ruminal microbes, and increased bacterial attachment and synergistic effects with hydrolysis of ruminal microorganisms (Colombatto et al. 2003).

This study was also designed to compare the efficacy of two methods of enzyme application. Some authors have suggested that pre-treatment of feed with enzymes could create a stable enzyme-feed complex (Kung et al. 2000), but others have indicated an alteration in the fiber structure, which would stimulate microbial colonization (Nsereko et al. 2000). In our study the FCE, FCR and ADF digestibility was affected more by application method. Several reports on animal response to feed enzymes mentioned that applying fibrolytic exogenous enzymes in a liquid form onto feeds prior to consumption could have a positive effect on animal performance (Rode et al. 1999; Yang et al. 2000). In contrast, direct infusion of enzyme into the rumen is far less effective or can even reduce digestibility of forage compared with application of liquid enzyme to substrate (Lewis et al. 1996).

In conclusion, supplemental EE can modify body weight gain of Pelibuey lambs and nutrient digestibilities without affecting nutrient intake, but the results of feed conversion efficiency and improved ADF digestibility depend on the application method used.
